# The Psychological Benefits of an Uncertain World: Hope and Optimism in the Face of Existential Threat

**DOI:** 10.3389/fpsyg.2022.749093

**Published:** 2022-03-23

**Authors:** Michael Smithson, Yiyun Shou, Amy Dawel, Alison L. Calear, Louise Farrer, Nicolas Cherbuin

**Affiliations:** ^1^Research School of Psychology, The Australian National University, Canberra, ACT, Australia; ^2^Centre for Mental Health Research, Research School of Population Health, The Australian National University, Canberra, ACT, Australia; ^3^Centre for Research on Ageing, Health and Wellbeing, Research School of Population Health, The Australian National University, Canberra, ACT, Australia

**Keywords:** hope, optimism, uncertainty, anxiety, depression, coronavirus

## Abstract

We examine how prior mental health predicts hopes and how hopes predict subsequent mental health, testing hypotheses in a longitudinal study with an Australian nation-wide adult sample regarding mental health consequences of the COVID-19 outbreak during its initial stage. Quota sampling was used to select a sample representative of the adult Australian population in terms of age groups, gender, and geographical location. Mental health measures were selected to include those with the best psychometric properties. Hypotheses were tested using generalized linear models with random intercepts, with the type of GLM determined by the nature of the dependent variable. Greater anxiety, depression, distress, and loneliness predict less hope, but impaired quality of life and stress *positively* predict hopes of gaining new skills. Distress and loneliness predict hopes for social connectedness and an improved society, suggesting that predictors of hope depend on what is hoped for. These findings suggest the need for more nuanced theories of hope. Greater hopes for societal improvement predict lower anxiety, depression, distress, and impaired quality of life, but greater hopes for skills and better mental health predict *higher* levels of these covariates. Moreover, when relevant prior psychological states are more intense, the impact of hope state declines. These findings indicate that the consequences of hope are heterogeneous, and suggest a possible explanation for the seemingly inconsistent therapeutic effectiveness of raising hope.

## Introduction

Uncertainty is often thought about as creating conditions for psychological distress. For instance, anxiety is often linked with fear of uncertainty, which has been proposed as a fundamental human fear (Carleton et al., 2012; Carleton, 2016). However, uncertainty is also a necessary pre-condition for some positive emotions, such as hope and optimism, and the resilience they afford (Smithson, 2008, p. 211). Thus, pandemic-induced uncertainty could also create conditions in which the psychological benefits of a positive future orientation through hope and optimism are amplified. This is important because positive emotions can help people to adopt a “broaden-and-build” stance (Fredrickson, 2004, 2013), even in the face of adversity. For instance, the hope and/or optimism components of curiosity, aspiration, and venturesomeness motivate acting on these orientations, thereby enhancing creativity and problem-solving abilities (Fredrickson, 2004).

To our awareness, longitudinal studies of the relationships between hope or optimism and various mental health covariates have been conducted under normal “everyday” conditions (e.g., Arnau et al., 2007). Ours is the first to do so under existential threat conditions, where these relationships become vitally important and where little is known about what forms they take. Unlike studies of hope and optimism where an individual is suffering from a disease (as in much of the medical literature), our study advances understanding of hope and optimism in the face of a population-level existential threat, which may strongly reduce hope and optimism in a population.

This article analyses responses to questions asking a representative Australian adult sample about their hope and optimism regarding outcomes arising from the COVID-19 outbreak, posed to the same respondents on two occasions in a seven-wave longitudinal survey focusing on the mental-health consequences of the outbreak. These data therefore offer an unusual opportunity for studying antecedents to and consequences of hope and optimism states during a crisis in a longitudinal data-set.

We begin by briefly reviewing the psychological functions and benefits of positive emotions in emotion regulation. We then focus on hope and optimism, examining their overlapping but distinct natures.

### Positive Emotions Broaden-and-Build

Fredrickson’s (2004, 2013) broaden-and-build model proposes that experiencing positive emotions broadens the repertoire of cognitive and behavioral actions available to the individual, and thereby builds resources for creativity and problem-solving. Positive emotions have immediate physiological and cognitive benefits. They counteract the physiological consequences of negative emotions, de-escalating arousal and returning the body to a relaxed state. Cognitively, positive emotions broaden attention and enable people to access a greater variety of cognitive and behavioral responses. This is the “broaden” part of Fredrickson’s model.

The “build” part proposes that broadened mindsets build enduring personal resources and capabilities, so that primary consequences of positive emotions include “post-consumption” cumulative benefits. These cumulative benefits extend to multiple domains, including resilience, coping, mental health, and social benefits (e.g., people who experience positive emotions also excel at eliciting positive emotions in others).

Of specific relevance to the present study, Fredrickson et al. (2003) presents evidence that positive emotions help build resilience against depressive responses in times of crisis. People’s reported experiencing of positive emotions after the 9–11 terrorist attack fully mediated the association between pre-attack resilience and both post-attack depressive symptoms and increases in psychological resources (life satisfaction, optimism, tranquility). Fredrickson (2013, p. 4) observes that hope differs from other positive emotions because hope can be experienced in both safe and dire circumstances. Feeling hope in a crisis motivates people to act against the crisis and find solutions to the problems that have generated it.

### The Nature of Hope and Optimism

Hope and optimism are closely related but non-redundant constructs, (e.g., Alarcon et al., 2013). Of the two, hope appears as the more contestable construct. Schrank et al. (2008) claim to find 49 definitions of it with 7 “dimensions” and 32 scales for measuring it. Nonetheless, three perspectives on hope have dominated the literature for three decades.

First, Snyder et al. (1991) define hope as constituting beliefs that a person has pathways to achieving a goal and the agency for accessing those pathways. Second, Herth (2000) presents a three-dimensional definition of hope, the first two dimensions bearing similarities to Snyder’s pathways and agency. Herth’s (2000) third affiliative-contextual dimension refers to a sense of possessing external support, mainly through belonging in social networks. Relatedly, Bernardo (2010) extends Snyder’s definition of hope to incorporate internal and external (e.g., interpersonal) loci.

Third, Averill et al. (2012/1990) define hope more inclusively by not limiting it to the achievement of personal goals. Moreover, they explicitly incorporate an affective component into their concept of hope, whereas the Snyder and Herth frameworks emphasize cognitive appraisal and neglect emotion. Finally, Averill et al. (2012/1990) emphasize the specificity of hope as a state (rather than as a trait), suggesting that understanding how hope works requires attention to what is hoped for and who will reap the benefits of the hoped-for outcome.

Similarly to hope, optimism has been conceptualized in several ways (Peterson, 2000; Carver et al., 2010). Scheier and Carver (1985) define optimism as an enduring personality characteristic or “disposition,” defined by the expectation of positive experiences in the future. The theoretical basis for dispositional optimism is that behavior is motivated by the pursuit of goals, which is influenced by the goal’s *value* – how important the goal is to a person, and *expectancy* – the confidence that a person has in attaining the goal.

Alternatively, Seligman (1991) conceptualizes optimism as an explanatory style, and argues that routinely optimistic and pessimistic explanations of events lead to different expectations about the future. Optimists attribute the causes of negative events to external, temporary and specific factors. That is, they believe that problems in life are fleeting in nature, caused by other people or situational factors, and are contained to a particular context. An optimistic explanatory style has been linked to enhanced physical well-being, motivation, achievement, and lower levels of depressive symptoms (for a review see Buchanan and Seligman, 1995).

There is a well-documented link between optimism and/or hope and various positive physical and psychological health outcomes (Scheier and Carver, 1992; Scheier et al., 2001; Carver et al., 2010; Alarcon et al., 2013). Positive expectations have been shown to increase well-being, decrease distress, enhance the speed of physical recovery and predict lower rates of rehospitalization in those undergoing medical procedures (Carver et al., 2010). Optimists and hopeful people tend to experience lower rates of depression and anxiety (Alarcon et al., 2013), and engage in behaviors that promote good physical health (Carver et al., 2010).

That said, the question of whether mental health variables predict hope or optimism remains partially unanswered. The longitudinal study focusing on hope reported by Arnau et al. (2007) indicated that neither anxiety nor depression predicts hope, although the hope pathways component did predict both of these mental health variables. There are several longitudinal studies of dispositional optimism, including a few investigating its trajectory over substantial portions of the lifespan. Chopik et al. (2015) report a 2-wave study in which they find that better health predicts increased optimism, and Schwaba et al. (2019) present evidence from a 4-wave study that experiencing positive life events increases optimism but negative life events do not appear to have an impact on optimism. However, in an examination of the impact of life events and changes on optimism using large-scale panel surveys from three countries, Chopik et al. (2020) report that these influences are not apparently homogeneous across the samples. In two samples, they observed that optimism was higher among middle-aged adults than among younger or elderly adults but this pattern did not emerge in their third (German) sample. Likewise, they found that positive and negative life events were inconsistently related to optimism across the three samples.

### Operationalizing Hope and Optimism

While the literature sometimes has treated hope and optimism separately, in the present study we believe there are several good reasons to consider them together, especially given that we will be measuring states rather than traits or dispositions. First, the features they have in common are of particular interest in the context of an existential crisis: Both are positive future-oriented emotions which arise under uncertainty.

Second, it can be difficult to extricate these two constructs operationally, especially in a survey relying solely on self-report. Laypersons often use the terms “hopeful” and “optimistic” interchangeably. For instance, Bury et al. (2016) found correlations of 0.46 and 0.60 between hope and optimism ratings in two studies.

Moreover, the literature lacks a systematic perspective on the relationship between these psychological states and uncertainty, but Bruininks and Malle (2005, p. 327) speculate that hope represents more important but less likely outcomes than what optimism represents. One may hope to win a million-dollar lottery but not be optimistic about winning it.

Relatedly, Bury et al. (2016, 2019) report that ratings of hope and optimism are distinguishable only when the probability of a desired outcome is low (with hope ratings exceeding those of optimism), but as this probability increases the ratings become indistinguishable. It was initially unclear to us whether everyone in our sample would have homogeneous views about the probabilities of the hoped-for outcomes that we targeted. Thus, using “hope” or “optimism” exclusively in our questionnaire items could potentially exclude or misrepresent some respondents’ expectations and feelings about such outcomes, as suggested by Bruininks and Malle (2005) and Bury et al. (2016). We therefore decided to include both terms, “hopeful” and “optimistic,” in our item questions.

We also found that published measures of hope or optimism did not suit our purposes, for two reasons. First, they reflect particular theoretical orientations which we considered too limiting, such as the restriction of hope to goal-oriented cognitive appraisal, as in Snyder’s, Herth’s, and Scheier and Carver’s frameworks. Some hoped-for outcomes in the COVID-19 crisis cannot constitute personal goals, such as the development of an effective vaccine or the cessation of government-imposed lockdown restrictions. Moreover, lockdown restrictions during the crisis have affected social contact and relations, highlighting the relevance of Bernardo’s (2010) external hope locus and Herth’s (2000) affiliative-contextual dimension. These considerations motivated us to adopt a perspective on hope akin to the Averill et al. (2012/1990, Ch. 5) framework. Likewise, the Life-Orientation Test (Scheier and Carver, 1985) is not sufficiently oriented toward hope and instead is widely regarded as a measure of optimism.

A second reason for not using published hope or optimism scales is that they do not attend to specific hopes relevant to the COVID-19 crisis, but instead are better suited to measuring dispositional hope or optimism. There is some recognition in the literature that what is hoped for can influence the role that hope plays in psychological adaptation, but even Sympson’s (1999) domain-specific dispositional hope scale was not suited to assessing hope states in this crisis (also, that scale is grounded in Snyder’s definition of hope). We therefore opted to create items targeting specific hopes relevant to the COVID-19 crisis. These are elaborated below.

### Role of Hope and Optimism in Psychological Functioning

Our study presents unique opportunities for extending understanding of the relationships between hope and/or optimism and mental-health and other psychological trait and state covariates. Because several relevant covariates were measured before, with, and after the hope-optimism items in the longitudinal survey, and because the hope-optimism items were measured twice, it is possible to examine the extent to which these covariates predict the hope-optimism item ratings and vice-versa.

A comprehensive meta-analysis presented by Alarcon et al. (2013) reports associations between a variety of psychological covariates and measures of hope and optimism. Optimism has been found to be associated with:

• Agency and pathways.
• Personality (including the five-factor traits: openness, conscientiousness, extroversion, agreeableness, and neuroticism).
• Psychological wellbeing (a large number of variables, including loneliness).
• Physical wellbeing.

Hope has been found to be associated with:

• Agency and pathways.
• Personality (but five-factor traits not tested).
• Psychological wellbeing (a more limited variety of variables).

Notably, physical wellbeing was not tested for association with hope in any of the studies in the collection.

The associations are in the expected directions. Thus, hope and optimism negatively covary with variables indicating or generating psychological distress or dysfunction, such as anxiety, depression, stress, and loneliness. Likewise, they positively covary with indicators or producers of psychological wellbeing, such as happiness, coping, self-esteem, and quality of life. However, the literature is not clear about whether or when hope or optimism are causes or consequences of these aspects of psychological wellbeing or distress. Also absent is a clear picture of the extent to which these associations depend on the nature of the desired outcome (e.g., whether optimism or hope has a similar impact on wellbeing regardless of whether the outcome is a personal goal or not).

### The Present Study

The research reported here was embedded in The Australian National COVID-19 Mental Health, Behaviour and Risk Communication (COVID-MHBRC) Survey, which was intended to examine the psychological impact of the COVID-19 pandemic on a representative sample of the Australian adult population (≥18 years). A multidisciplinary team (epidemiology, mental health, psychology, and statistical methods) designed the study, which was issued in seven online survey waves fortnightly starting on 28 March 2020, using the Qualtrics survey platform. Our study was approved by The Australian National University Human Research Ethics Committee (protocol 2020/152). The full study protocol is available in the Supplementary Material provided by Dawel et al. (2020).

Prior to the 2019–2020 bushfires and the COVID-19 outbreak, Australia had enjoyed decades of social and economic stability, e.g., among the countries least affected by the 2008 global financial crisis. The combination of extremely severe bushfires and the arrival of COVID-19 was a strong shock and raised challenges of magnitudes not seen for generations. Nonetheless, among Western developed countries, the Australian response to COVID-19 was one of the most successful in containing and suppressing the virus, thereby creating conditions where hopes would be more likely to arise than in many other countries. We therefore expected to find evidence of hopefulness among participants.

The contents of our hope-optimism items were determined by two influences in addition to the literature on hope and optimism. First, our choices were based on themes emerging from journalists’ interviews with members of the public and advice from medical experts about how best to respond to the crisis and what outcomes would emerge. These had to be outcomes that were uncertain, i.e., neither inevitable nor impossible. They also had to be outcomes for which hope would be regarded as a valid and justifiable feeling. The criteria we applied were personal relevance, relative importance, and moral legitimacy (e.g., ruling out hopes that a despised politician would contract the disease).

Second, given that space was scarce in the survey (whose primary focus was on mental health), we were constrained to a limited number of items. A crucial choice was between items referring to approaching positive end-states versus avoiding negative end-states. Positive end-states include both personal goals such as improving one’s mental health and generalized states such as a more egalitarian society. Negative end-state examples are avoiding getting COVID-19 and preventing economic collapse.

We chose positive end-state hopes for two reasons. First, hope and optimism both serve as affective-cognitive components of self-regulation. Most theories of self-regulation distinguish between the motive to approach positive end-states and the motive to avoid negative end-states (e.g., Atkinson, 1964; Carver and Scheier, 1981; Bandura, 1986; Higgins, 1998). There is a general view in these theories that a focus on achieving positive end-states yields greater and more durable psychological benefits (Higgins, 1989, 1998). Second, many of the questions in our survey understandably emphasized attitudes, beliefs, and actions regarding avoidance and suppression of COVID-19. There were relatively few items tapping attitudes about desired end-states.

We elected to measure hope and optimism related to three positive personal goals and one generalized end-state:

• Gaining new abilities or skills—self-efficacy, resilience, adaptability.
• Improved mental health—resilience, coping.
• Greater connectedness with family and/or friends—social support, belonging.
• Improved society—A society emerging that has improved in one or more ways.

The first two goals arguably correspond with Bernardo’s (2010) internal locus, whereas social connection and societal improvement are more external. These items were measured in waves 2 and 5, using a four-point scale (Table 2). In wave 5, we also asked participants to rate how negatively or positively they anticipated the consequences of the COVID-19 crisis would be in seven aspects of their lives: privacy, legal restrictions, adaptation, coping, skills, relationships, and recreation. While these items are not direct measures of hopefulness or optimism, they provide an alternative measure of participants’ future orientations as influenced by the crisis.

The survey provided numerous covariates that may be associated with hope/optimism, including variables regarding the impact of COVID-19 and responses to it. It is important to bear in mind that this survey had to serve multiple purposes of the research team, so there were constraints on the extent to which it could be shaped for this particular investigation. That said, several among the types of covariates that have been ascribed associations with hope and optimism were included in the survey, so our study is able to test such claims about relationships between hope and optimism and other psychological variables. The longitudinal data enable investigations about what predicts hope/optimism, and what hope and optimism predict about psychological distress and social functioning. We also can investigate the specificity of these predictions. Hope or optimism about skills and abilities, mental health, and social connection may differentially predict and be predicted by covariates in each of those domains.

Finally, we can investigate whether these predictive relationships seem stable across wave-pairs, because successive waves are identically spaced in time (i.e., a fortnight apart). Given the rapidly changing circumstances of the crisis, this will provide a relatively severe test of stationarity in these relationships as well as an evaluation of their sensitivity to changing conditions. Why is this important? Lawlike relationships do not change over time or from one context to another. A test of stationarity is a test of a necessary (though not sufficient) condition for determining whether a relationship is lawlike.

#### Hypotheses and Exploratory Questions

Two groups of hypotheses were tested, along with related exploratory questions: Which variables predicted the hope-optimism (HO) items and which variables were predicted by the HO items. These variables are denoted by generic terms in the following list, whereas the scales measuring them are described in the section “Materials and Methods.” Hypotheses and investigations in the first group included the following.

1. The following covariates will positively predict HO, especially in the skills and adaptability items.

a. Wellbeing.
b. Coping.

2. Sense of belonging will positively predict the two HO social items.
3. The following covariates will negatively predict all HO items.

a. Anxiety.
b. Depression.
c. Distress.
d. Stress.
e. Loneliness.
f. Lack of agency.
g. Impaired quality of life.

4. Prior psychological or medical trauma will negatively predict all HO items.
5. Higher education will positively predict HO, especially in the Skills and Mental Health items.
6. Demographic variables in wave 1 included age, gender, and education level. There were no hypotheses regarding the effects of gender and age on HO item ratings, but they were included with education in all models.

Hypotheses and investigations in the second group were as follows:

7. HO items will predict the following covariates:

a. Anxiety (negatively).
b. Depression (negatively).
c. Distress (negatively).
d. Loneliness (negatively).
e. Impaired quality of life (negatively).
f. Stress (negatively).
g. Coping (positively).

8. HO items will positively predict ratings of anticipated outcomes:

a. The Skills HO item will positively predict the Adaptation, Coping, Skills, and Recreations outcome items.
b. The Mental Health HO item will positively predict the Adaptation, Coping, Relations, and Recreations outcome items.
c. The Connectedness HO item will positively predict the Adaptation, Coping, and Relations outcome items.
d. The Societal Improvement HO item will positively predict the Adaptation, Coping, and Relations outcome items.

## Materials and Methods

Quota sampling was employed to obtain a representative sample of 1,296 participants on the basis of age group, gender, and geographical location (State/Territory). Details of the rationale for the choice of the first-wave sample size are available in Dawel et al. (2020). Participants received up to five reminders for the next survey within the week following a completed wave. The first wave was conducted during the last week of March 2020 (28–31 March), and the waves were spaced approximately fortnightly apart, with the seventh and final wave collected at the end of June 2020 (seven waves).

The spacing and number of waves were largely determined by practical considerations regarding the first wave of the virus in Australia and the multiple purposes of the research team. During this period in Australia non-essential businesses were shut down, Australians were encouraged or required to work from home and practice social distancing when out in public, they were forbidden to travel overseas, and several state borders were closed across the period of the survey. At the time, no-one knew how long the pandemic might last, but developments were happening very rapidly. Adequately recording the trajectories of mental health indicators during these developments clearly required more than just two or three waves, but we also were mindful of how difficult it might be to retain participants over multiple waves. We settled on seven waves based on team members’ prior experience longitudinal health surveys and information about participant attrition rates from the relevant literature.

Sample attrition initially was 27% from wave 1 to wave 2 but thereafter less than 10% per wave. Sample sizes in subsequent waves were W2 = 969, W3 = 952, W4 = 910, W5 = 874, W6 = 820, and W7 = 762. The hope items were measured in W2 and W5, so the waves predicting them are W1 ad W4, and the waves containing covariates predicted by them are W3 and W6. Thus, the analyses in this article utilize data from the first six waves because the 7th wave is not relevant to our topic.

Table 1 lists the relevant variables, and the relevant waves in which they were measured. Symptoms of depression and anxiety were assessed by the Patient Health Questionnaire-9 (Spitzer et al., 1999) and Generalized Anxiety Disorder-7 (Spitzer et al., 2006), respectively. Psychological distress was measured by the Distress Questionnaire-5 (Batterham et al., 2016). Impairment to quality of life was measured using a 9-point scale (from “not at all impaired” to “very seriously impaired”), on five aspects: Ability to work, home management, social leisure activities, private leisure activities, and maintenance of relationships. General psychological well-being was measured using the World Health Organization Well-Being Index (Topp et al., 2015). Sense of belonging was measured by the Interpersonal Needs Questionnaire (INQ) scale (Van Orden et al., 2012), and loneliness was measured over the previous 2 weeks with the 6-item De Jong Gierveld Loneliness Scale (de Jong Gierveld and van Tilburg, 2006). Coping ability and stress level were measured by single-item ratings for the past 2 weeks (on 6-point scales from “not at all” to “extremely”). Lack of agency was measured by the Pearlin Mastery Scale (Pearlin and Schooler, 1978).

**TABLE 1:** Relevant constructs and variables.

Construct	Variables	As DV: Type of GLM	Waves predicting HO items	Waves predicted by HO items
Hope/optimism	Four rating items	Ordinal	NA	2, 5
Lack of agency	Pearlin Mastery Scale		4	NA
Demographics	Gender, age, education		1	NA
Anxiety	Generalized Anxiety Disorder-7	Tweedie	1, 4	2, 3, 5, 6
Depression	Patient Health Questionnaire-9	Tweedie	1, 4	2, 3, 5, 6
Distress	Distress Questionnaire-5	Tweedie	1, 4	2, 3, 5, 6
Coping level	One rating item	Ordinal	1, 4	2, 3, 5, 6
Stress level	One rating item	Ordinal	1, 4	2, 3, 5, 6
Loneliness	De Jong Gierveld Loneliness Scale	Gaussian	1, 4	2, 3, 5, 6
Belonging	INQ scale		1	NA
General wellbeing	World Health Organization Well-Being Index		1	NA
Prior psychological problems	Count of checklist		1	NA
Prior physical or medical problems	Count of checklist		1	NA
Impaired quality of life	Sum of five rating-items	Tweedie	1, 4	2, 3, 5, 6
Future outcome ratings	Five rating items	Ordinal	NA	5

Relevant demographic and background variables included in our analyses are age (in years), gender (male/female/other, the four cases of “other” were omitted), years of education, and existing or prior health, neurological or psychological conditions as diagnosed by an appropriate clinician (count of items ticked on checklists). Gender, age, education level, prior medical and psychological issues all were controlled for in the models described below to take into account any effects of changes in their compositions due to sample attrition.

### Analytical Strategies

Where possible (e.g., in mixed GLMs), the full sample from each wave was used; otherwise the relevant sample consisted of the latest wave in the analysis. Relevant demographic summary statistics are in Supplementary Table 1. Hypotheses in both groups were tested using generalized linear models (GLMs) with random intercepts, with the type of GLM determined by the nature of the dependent variable. The HO item prediction models were conducted using mixed ordinal logistic regression. The models with HO items predicting covariates employed compound Poisson-gamma (Tweedie) distribution, normal distribution, or ordinal logistic GLMs, depending on the covariate distributions (as listed in Table 1, with further details in the section “Results”).

Both groups of GLMs included models for two pairs of waves. The HO-prediction models had predictors in waves 1 and 4 predicting HO items in waves 2 and 5, respectively; while the HO-predicting models had HO items in waves 2 and 5 predicting covariates in waves 3 and 6, respectively. We treated the HO item ratings as a collection of four categorical ordinal variables. The HO-prediction models were developed initially by entering each candidate covariate into a model that already included wave 1 demographics and background variables: gender, age, education, a count of prior medical issues, and a count of prior psychological issues.

The HO-predicting models tested the effects of the HO items on the mental health covariates listed in Hypothesis 7, and on the outcome variables listed in Hypothesis 8. Covariates controlled for in these models as mentioned earlier included gender, age, education level, prior medical and psychological issues, and the appropriate mental health covariate score in the prior wave. Supplementary Table 1 shows that changes in the compositions of these covariates were quite small.

As foreshadowed earlier, a key model-comparison that is not in the hypotheses listed above, is between a model that restricts the coefficients for both pairs of waves to be identical and a model relaxing that restriction, allowing wave-pair to moderate the coefficients. This is a test of stationarity. For instance, if the coefficients for a model using covariates in wave 1 to predict HO items in wave 2 are the same as the coefficients for the same covariates in wave 4 predicting HO items in wave 5, that constitutes evidence for the stationarity of those covariate-HO relations during the course of the COVID-19 crisis. This test is appropriate because successive waves were nearly equally separated by a fortnight, although we must bear in mind that the dynamics of these relations cannot be ascertained with just two time-points. We note that the longitudinal study reported by Arnau et al. (2007) assumed stationarity in their structural equations model, but did not test it.

## Results

### Dependent Variable Distributions

This section briefly reviews the distributions of the dependent variables in subsequent analyses. We begin with the HO items, followed by the outcome ratings and the mental health covariates.

Despite COVID-19, some participants registered hopefulness and anticipated positive outcome possibilities resulting from the crisis and their ways of coping with it. Table 2 shows the percentage distributions in waves 2 and 5 of ratings of the four HO items. There was a slight decline from wave 2 to 5 in the Connectedness and Societal Improvement items, but the distributions are mostly similar across the waves. Participants were most hopeful about becoming more socially connected and for society to emerge improved in some respect, with nearly half of the sample expressing at least moderate hopefulness regarding those outcomes.

**TABLE 2:** Hope-optimism item distributions for waves 2 and 5.

	New skills		Mental health		Social connectedness		Societal improvement	
Correlation	0.505		0.412		0.471		0.479	

Degree of hope	wave 2	wave 5	wave 2	wave 5	wave 2	wave 5	wave 2	wave 5

Not at all	48.7%	48.1%	38.5%	33.8%	24.3%	24.1%	15.9%	16.6%
Slightly	30.0%	31.2%	28.0%	36.3%	28.3%	29.7%	28.3%	33.6%
Moderately	14.6%	13.9%	21.9%	19.9%	27.0%	28.2%	31.8%	31.2%
Very hopeful	6.7%	6.8%	11.6%	10.0%	20.4%	17.9%	24.0%	18.6%
Sample size	803	797	803	797	803	797	803	797

Although reliability is not directly measurable here, and despite the fact that we would expect differences in individuals’ changes in hope levels from wave 2 to 5 due to differential influences from covariates, we still can indirectly address reliability in two ways. The simplest indicator is the wave 2–5 Kendall tau correlations, which are shown in the top row of Table 2, ranging from 0.412 to 0.505. The second way is in the test of stationarity mentioned earlier and described below. A test of stationarity is stronger evidence than tests of reliability, as it is exceedingly unlikely that unreliably measured variables will have stable covariations among each other over time.

We treated the wave 5 outcome ratings as categorical ordinal variables. Table 3 displays the percentage distributions of the participants’ ratings regarding seven anticipated outcomes or consequences of the COVID-19 crisis. The most negatively rated consequences are for the Legal and Recreation items. However, the other five items have greater percentages of positive than negative ratings, and a majority of ratings falling in the somewhat positive or positive categories for the Adaptation, Coping, and Skills items.

**TABLE 3:** Anticipated positive vs. negative outcomes distributions.

	Privacy	Legal	Adaptation	Coping	Skills	Relations	Recreation
Negative	2.6%	8.6%	1.4%	1.8%	0.6%	2.7%	13.6%
Somewhat negative	9.2%	35.4%	11.0%	10.8%	5.8%	11.8%	30.7%
Neutral	64.3%	35.5%	35.6%	30.8%	42.5%	38.9%	28.5%
Somewhat positive	14.0%	13.8%	33.6%	35.1%	31.6%	26.0%	18.3%
Positive	10.0%	6.7%	18.4%	21.6%	19.4%	20.7%	8.9%

The mental health dependent variables include anxiety, depression, distress, loneliness, and impaired quality of life. These were treated as quantitative continuous variables. Supplementary Figure 1 displays histograms for each of them in waves 3 and 6, showing that their distributions are similar in both waves. As typically happens with normal-functioning adults, the modal scores on anxiety, depression, distress, and impaired quality of life are at the bottoms of their respective scales. These are likely to be true scores, not censored scores in the technical sense (Smithson and Shou, 2019, p. 3–5, 29). We therefore elected to model these covariates via GLMs using compound Poisson-gamma (Tweedie) distributions because those distributions explicitly model the “zeros” (the cases on the boundary) in scales with a lower bound. The exception is loneliness, which has a distribution shape suitable for normal-theory regression.

### Predictors of Hope-Optimism Items

#### Stationarity Tests

Evidence for stationarity was obtained for most of the covariates in the ordinal logistic regression models. Of the wave 1 demographic and background variables, all of their effects were moderated by HO item, but only the prior medical and psychological problems variables had item-wave moderator effects, indicating that they had different effects on HO items in waves 2 and 5. Importantly, effects of the two background psychological covariates, general wellbeing and belonging (both at wave 1), were moderated only by HO item. A model that included a wave effect for these two covariates as well as their moderation by HO item did not significantly improve fit over a model including only moderation by HO item (χ_8_^2^ = 4.87, *p* = 0.771).

The crucial stationarity tests, however, were those comparing the loneliness, distress, anxiety, depression, impaired quality of life, stress, and coping effects from wave 1 to 2 with their counterparts from wave 4 to 5. There were no significant moderator effects from wave-pair for any of these covariates except for stress. Even for stress, the wave effect was a main effect only, not one that further depended on the type of HO item. Further details are provided below.

#### Hypothesis 1

The covariates hypothesized to positively predict HO items included wellbeing, and coping. Wellbeing was measured at wave 1, and its effect was moderated only by HO item and not by wave, thereby suggesting that its effects are stationary. In all models wellbeing significantly positively predicted Skills and Mental Health but not Connectedness or Societal Improvement (further details are available in Supplementary Table 4). Hypothesis 1a therefore received partial support.

Coping was measured at waves 1 and 4. A model with a main effect for coping yielded a significant positive effect [*z* = 4.12, *p* < 0.0005; odds-ratio = 1.119, 95% CI = (1.061, 1.180)]. A model including moderator effects from HO item and wave did not significantly improve fit (χ_4_^2^ = 4.65, *p* = 0.325). Hypothesis 1b therefore was supported.

#### Hypothesis 2

The belonging (INQ) measure was assessed at wave 1, and as mentioned above, its effect was moderated only by HO item and not by wave, thereby suggesting that its effects were stationary. In all models it significantly positively predicted all HO items (*z* ≥ 2.92, *p* ≤ 0.005), most strongly for the Connectedness and Societal Improvement items, as anticipated in Hypothesis 2. Details are available in Supplementary Table 4.

#### Hypothesis 3

We now focus on the covariates that were hypothesized to negatively predict HO item ratings. The main findings are briefly described here; details are available in Supplementary Tables 5–7.

Anxiety had a significant negative main effect [*z* = −2.66, *p* = 0.008; odds-ratio = 0.972, 95% CI = (0.953, 0.993)]. A model with HO item as moderator of the anxiety effect did not significantly improve fit over the main-effect model (χ_3_^2^ = 6.23, *p* = 0.101), and a model with wave as a moderator did not significantly improve fit (χ_1_^2^ = 1.77, *p* = 0.183). The anxiety effect therefore appeared stationary and unmoderated by HO item. Hypothesis 3a was supported.

Depression also had a significant main effect (χ_1_^2^ = 11.85, *p* = 0.001). A model with HO item as moderator of the depression effect significantly improved fit over the main-effect model (χ_3_^2^ = 9.57, *p* = 0.023), but a model with wave as a moderator did not significantly improve fit (χ_1_^2^ = 0.58, *p* = 0.447). The depression effect therefore appeared stationary and was moderated by HO item. The significant effects were negative for Mental Health (*z* = −2.25, *p* = 0.025), Connectedness (*z* = −3.64, *p* < 0.0005), and Societal Improvement (*z* = −3.67, *p* < 0.0005), whereas Skills was not significant (*z* = −1.27, *p* = 0.205). Hypothesis 3b received partial support.

Distress exhibited significant main and HO item moderator effects (χ_4_^2^ = 23.22, *p* < 0.0005). A wave-by-item model did not significantly improve fit (χ_3_^2^ = 1.51, *p* = 679). The distress effect could be regarded as stationary and moderated by HO item. The distress effect was negative for Connectedness (*z* = −2.51, *p* = 0.012) and Societal Improvement (*z* = −2.05, *p* = 0.041), but the effects were non-significant for Skills (*z* = −0.60, *p* = 0.550) and Mental Health (*z* = −0.28, *p* = 0.781). Hypothesis 3c received partial support.

Stress effects exhibited significant moderation by HO item (χ_4_^2^ = 12.56, *p* = 0.014) and by wave (χ_1_^2^ = 4.79, *p* = 0.028), but with no significant wave-by-item effect (χ_3_^2^ = 3.26, *p* = 0.354). Thus, the effects identified for stress are non-stationary and heterogeneous across the HO items. However, wave 1 stress negatively predicted the wave 2 Societal Improvement item (*z* = −2.45, *p* = 0.014) whereas wave 4 stress positively predicted wave 5 Skills (*z* = 2.07, *p* = 0.038). None of the other effects for wave 2 or wave 5 HO items were significant (| *z*| ≤ 1.50, *p* ≥ 0.135). Thus, hypothesis 3d was not supported.

Loneliness had a significant main effect (χ_1_^2^ = 38.68, *p* < 0.0005). A model with HO item as moderator of the loneliness effect significantly improved fit over the main-effect model (χ_3_^2^ = 29.00, *p* < 0.0005), but a model with wave as a moderator did not significantly improve fit (χ_1_^2^ = 0.12, *p* = 0.724). The loneliness effect therefore appeared stationary and was moderated by HO item, although it turned out that its effects were significantly negative for all HO items, most strongly so for Connectedness and Societal Improvement. Hypothesis 3e was supported.

The Pearlin Mastery scale had a significant main effect (χ_1_^2^ = 39.48, *p* < 0.0005). A model with HO item as moderator of the lack of agency effect significantly improved fit over the main-effect model (χ_3_^2^ = 9.12, *p* = 0.028). Lack of agency scores negatively predicted all HO items, most strongly for Connectedness and Societal Improvement. Hypothesis 3f was supported.

Impaired quality of life effects with HO item as moderator significantly improved fit over a main-effect model for impaired quality of life (χ_3_^2^ = 32.60, *p* < 0.0005), but a model with wave as a moderator did not significantly improve fit (χ_1_^2^ = 1.39, *p* = 0.239). The impaired quality of life effect therefore appeared stationary and was moderated by HO item. Contrary to hypothesis, impaired quality of life significantly positively predicted Skills (*z* = 4.51, *p* < 0.0005), but did not significantly predict Mental Health, Connectedness, or Societal Improvement (*z* ≤ 1.61, *p* ≥ 0.108). Hypothesis 3g was not supported.

#### Hypothesis 4

Prior medical and psychological issues had little impact on the HO items, although as mentioned earlier, their effects seemed to be non-stationary. Nevertheless, their effects were small and inconsistent across items. Thus, hypothesis 4 did not receive support. Additional details are available in the Supplementary Material.

#### Hypothesis 5 and Demographics (6)

Gender made no significant independent contribution to predicting the HO items. Age had a negative effect on the Skills item [*z* = −8.23, *p* < 0.0005; odds-ratio = 0.946, 95% CI = (0.934, 0.959)] and Mental Health [*z* = −4.48, *p* < 0.0005; odds-ratio = 0.976, 95% CI = (0.966, 0.986)] but had a marginal positive effect on the Societal Improvement item [*z* = 2.17, *p* = 0.030; odds-ratio = 1.021, 95% CI = (1.001, 1.024)]. Education had a significant positive effect on the Skills item [*z* = 2.74, *p* = 0.006; odds-ratio = 1.317, 95% CI = (1.082, 1.604)]. Hypothesis 5 received partial support. Additional details are available in Supplementary Table 3.

### Covariates Predicted by Hope-Optimism Items

#### Stationarity Tests

The models in this section assess the prediction of mental-health covariates in waves 3 and 6 with HO items in waves 2 and 5. The models are autoregressive order 1 in the sense that they include the corresponding covariate measured in waves 2 and 5. All covariates except for loneliness were fitted with random-intercept compound Poisson-gamma (Tweedie) distribution GLMs, with loneliness fitted with a random-intercept normal distribution model. Evidence for stationarity in the HO item effects was obtained for all of the covariates (see below). However, stationarity was not always observed in the autoregressive component of the models.

#### Hypothesis 7

Anxiety required an anxiety-by-wave model but showed stationarity because a model including wave-by-HO-item terms did not significantly improve model fit over a model without this term (χ_4_^2^ = 3.462, *p* = 0.484). Two of the anxiety-by-HO-item terms were significant but had opposite signs. The Skills HO item more weakly positively predicted anxiety as prior anxiety increased, whereas the Societal Improvement item more weakly negatively predicted anxiety as prior anxiety increased. The Mental Health and Connectedness items did not have significant effects. Hypothesis 7a was largely unsupported. More details are available in Supplementary Table 9.

Depression required a wave-by-depression model, but showed stationarity in the HO-item effects because a model including wave-by-HO-item terms did not significantly improve model fit over a model without this term (χ_4_^2^ = 1.779, *p* = 0.776). All of the depression-by-HO-item terms were significant but did not have the same signs. The Skills and Mental Health HO items’ coefficients for depression decreased as prior depression increased, whereas the Connectedness and Societal Improvement items’ coefficients for depression increased as prior depression increased. The effects were relatively small, but the Mental Health item generally positively predicted depression while Connectedness and Societal Improvement tended to negatively predict depression for low values of prior depression. Hypothesis 7b was largely disconfirmed. More details are available in Supplementary Table 10.

Distress did not require a distress-by-wave model (χ_1_^2^ = 2.173, *p* = 0.141), and showed stationarity because a model including wave-by-HO-item terms did not significantly improve model fit over a model without them (χ_4_^2^ = 3.556, *p* = 0.469). The Skills HO item more weakly positively predicted distress as prior distress increased, whereas the Societal Improvement item more generally negatively predicted distress. The Mental Health and Connectedness items did not have significant effects. Hypothesis 7c was largely disconfirmed. More details are available in Supplementary Table 11.

Loneliness did not require a loneliness-by-wave model (χ_1_^2^ = 1.368, *p* = 0.242), and showed stationarity because a model including wave-by-HO-item terms did not significantly improve model fit over a model without them (χ_4_^2^ = 8.410, *p* = 0.078). The Skills and Connectedness items more weakly positively predicted loneliness as prior loneliness increased. The Mental Health and Societal Improvement items did not have significant effects. Hypothesis 7d was disconfirmed. More details are available in Supplementary Table 12.

Impaired quality of life required an impaired quality of life-by-wave model, but showed stationarity for the HO-item effects because a model including wave-by-HO-item terms did not significantly improve model fit over a model without them (χ_4_^2^ = 3.175, *p* = 0.529). The Mental Health HO item negatively predicted impaired quality of life. The other three HO items had significant impaired quality of life-by-HO-item terms. The Skills and Connectedness items positively predicted impaired quality of life, but more weakly as prior impaired quality of life increased, whereas the Societal Improvement item negatively predicted impaired quality of life, but more weakly as prior impaired quality of life increased. Hypothesis 7e was largely disconfirmed. More details are available in Supplementary Table 13.

Stress was not significantly predicted by HO items (χ_4_^2^ = 0.646, *p* = 0.958), whereas Coping was significantly predicted by the HO items (χ_4_^2^ = 27.889, *p* < 0.0005). The latter relationship was not significantly moderated by wave (χ_4_^2^ = 1.338, *p* = 0.855) and therefore appeared stationary. However, it was moderated by prior coping level (χ_4_^2^ = 26.590, *p* < 0.0005). Coping was positively predicted by the Skills (*z* = 2.513, *p* = 0.012) and Societal Improvement item (*z* = 2.691, *p* = 0.007), but not significantly by the other HO items (*z* ≤ 0.966, *p* ≥ 0.334). Moderator effects from prior coping were significantly negative for both Skills (*z* = −2.124, *p* = 0.034) and Societal Improvement item (*z* = −2.073, *p* = 0.038). Hypothesis 7f was disconfirmed, whereas Hypothesis 7g received partial support. Further details are available in Supplementary Table 8.

#### Hypothesis 8

The consequence item ratings in wave 5 were predicted by the HO items in wave 2 with gender, age, and education (in wave 1) controlled for. A wave-by-HO-item terms significantly improved model fit over a model without this term (χ_4_^2^ = 85.891, *p* < 0.0005). The Skills HO item positively predicted all of the consequence item ratings (*z* ≥ 3.15, *p* < 0.002). The Mental Health item positively predicted the recreation consequence item (*z* = 3.13, *p* = 0.002); the Connectedness item positively predicted the adaptation (*z* = 2.09, *p* = 0.037), skills (*z* = 2.12, *p* = 0.034), and relationships (*z* = 5.09, *p* < 0.0005) consequence items; and the Societal Improvement item positively predicted the coping consequence item (*z* = 2.27, *p* = 0.023). Hypothesis 8a was supported; whilst 8b, 8c, and 8d received partial support. More details (including odds-ratios and 95% confidence intervals) are available in Supplementary Table 14.

## Discussion

This concluding section covers four topics: what predicts hope and optimism states, what hope and optimism predict, whether these relationships may be stationary, and implications of our findings for theoretical developments and future research. In the first two topics we focus on interpreting the outcomes of hypothesis tests and unexpected findings, the latter emerging as important in understanding what hope and optimism predict. A reconsideration of the stationarity tests addresses their generality and whether artifact may be involved.

### What Predicts Hope-Optimism Items

Many of the relationships between the mental health covariates and the HO items were in the expected directions, but there were some noteworthy exceptions. Moreover, the correlations among many of these covariates raised some interpretive problems that are relevant to both theory and practical applications. Table 4 contains a schematic summary of significant predictors of hope-optimism items.

**TABLE 4:** Summary of significant predictors of hope-optimism items.

Covariate	New skills	Mental health	Social connect.	Soc. improve.
Wellbeing	+	+		
Belonging	+	+	+	+
Coping	+	+	+	+
Anxiety	−	−	−	−
Depression		−	−	−
Distress			−	−
Stress	+ (wave 5)			− (wave 2)
Loneliness	−	−	−	−
Impair. qu. life	+			
Lack of agency	− (wave 5)	− (wave 5)	− (wave 5)	− (wave 5)

*Significant positive effects are denoted by + and negative effects are denoted by −.*

To begin, WHO wellbeing scores at wave 1 significantly positively predicted Skills and Mental Health but not Connectedness or Societal Improvement in waves 2 and 5. Coping at waves 1 and 4 positively predicted all of the HO items at waves 2 and 5, respectively. Lack of agency scores negatively predicted all HO items, most strongly for Connectedness and Societal Improvement. These findings point to the “other side of the coin” regarding Fredrickson’s (2004) broaden-and-build model. Possession of resources such as wellbeing and self-efficacy enable hope and optimism to be effective builders, and specific kinds of resources are enablers for specific objects of hope.

Belongingness at wave 1 also positively predicted all HO items, most strongly for the Connectedness and Societal Improvement items. Loneliness negatively predicted all HO items, most strongly for the Connectedness and Societal Improvement items. These relationships are in the expected directions, and they reflect the correlations among wellbeing, belonging, and loneliness. The stronger relations with the Connectedness and Societal Improvement items reinforce the importance of Herth’s (2000) affiliative-contextual dimension (a sense of external social support).

Turning now to indicators of mental health, the effects of anxiety and depression on hopefulness were negative as expected. It should be noted that the correlation between anxiety and depression was 0.860, so it is no surprise that their effects are similar. Likewise, psychological distress negatively predicted the Connectedness and Societal Improvement items, although it did not significantly predict Skills or Mental Health. These findings differ from the longitudinal study reported by Arnau et al. (2007), who did not find that anxiety or depression predicted hope. However, their study used American undergraduates, measured dispositional hope with the Snyder et al. (1991) hope scale, and was not oriented to an existential threat.

More difficult to interpret are the effects of stress and impaired quality of life. First, impaired quality of life *positively* predicted Skills and wave 4 stress positively predicted the wave 5 Skills item (although this latter finding did not hold for wave 1 predicting wave 2). Both findings are contrary to our hypotheses. One interpretation is that decrements to quality of life are being treated as challenges to be overcome by gaining new skills. Another possibility is that the restrictions impairing quality of life nonetheless opened up opportunities and leisure time to learn new skills and connect with family or friends. We return to these interpretations in the concluding section.

### What Hope-Optimism Items Predict

Almost none of the hypotheses in this set were supported, and to some extent the findings were opposite to expectations. The “internal” HO item Skills tended to positively predict anxiety, depression, distress, and impaired quality of life, whereas the “external” HO item Societal Improvement generally negatively predicted these covariates. On the other hand, the Skills and Connectedness items both positively predicted loneliness. Finally, the Skills and Societal Improvement items positively predicted coping, as expected.

Two interpretive issues present themselves here. First, the finding that higher scores on hope-optimism regarding skills predicted poorer mental health appears to go against Fredrickson’s broaden-and-build theory. The same is true of Connectedness scores positively predicting subsequent loneliness scores. A possible explanation is that higher hope or optimism renders a person more vulnerable to their hopes not eventuating, resulting in a downturn in their psychological states. Participants were likely to find evidence regarding whether their goals were being met soon after being asked to rate hopes for matters such as skill development, but not so for a hope unrelated to goals such as societal improvement. If so, then this imposes a scope condition on the broaden-and-build perspective: Disappointment may undo the broaden-and-building effect of hope.

Second, contrary to the view presented by unconditional associations between hope or optimism and psychological covariates reported throughout the relevant literature [e.g., as summarized in Alarcon et al. (2013)], what hope or optimism predicts for other psychological covariates appears to be conditioned by two things. First, these associations are moderated by the prior state of the covariate. They are stronger when prior scores on the covariates are lower, i.e., when the psychological states they are measuring are less intense. As those states intensify, the impact of hope and optimism declines. For the extremely skewed covariates (anxiety, depression, distress, impaired quality of life) the maximum effects of hope apply to the approximately 25–33% of the sample whose prior scores are at the bottom of the scales, but this is not the case for a symmetrically distributed covariate such as loneliness.

Additionally, the associations depend on what is hoped for, underscoring the importance of Bernardo’s (2010) internal-external distinction. The “internal” Skills and “external” Societal Improvement items exhibited *opposite directions* in their predictions of mental health covariates.

Lastly, hypothesis 8 received partial support. There was some separation between the internal and external HO items’ effects, although Skills HO item positively predicted all of the consequence item ratings. The Mental Health item positively predicted the recreation consequence item, the Connectedness item positively predicted the relationships item, and the Societal Improvement item positively predicted the coping item. These findings generally support Fredrickson’s broaden-and-build perspective. Hope and optimism lead to a more positive orientation toward future prospects, even under duress. More specifically, hope and optimism regarding personal outcomes of a crisis may generate more positive predictions about personal consequences of that crisis.

### The Question of Stationarity

As indicated earlier, evidence for stationarity in relationships between variables is evidence that those relationships may be stable. In this study it also amounts to replication in a test-retest sense. Stationarity has been indicated by the results in most of the models testing both sets of hypotheses in this article. It is reasonable to ask whether the apparent “stationarity” could be due to artifact in the data. Prime candidates for this would be strong autocorrelation in the hope items and/or invariance in HO items across the waves. Starting with the question of invariance, 41.2% of the Skills ratings changed from wave 2 to wave 5, as did 50.2% of the Mental-Health ratings, 52.8% of the Connectedness ratings, and 52.8% of the Societal Improvement ratings. Thus, low variance in the data does not account for stationarity. Turning to autocorrelation, Kendall’s tau correlations between wave 2 and wave 5 HO item ratings were 0.505, 0.412, 0.471, and 0.479, which are not strong enough to account for stationarity on their own.

The evidence of stationarity in the relationships between hope-optimism and other psychological covariates is rather remarkable, given the tumultuous nature of a crisis such as COVID-19. Nevertheless, this evidence should be treated with caution. Two measurements in a longitudinal study cannot reveal the nature of the dynamics underpinning these relationships. It also is worth bearing in mind that this study took place before the second-wave and subsequent outbreaks in the most populous parts of Australia, which led to a significant increases in cases, deaths and social distancing restrictions as well as shut-downs.

### Conclusion and Future Directions

We conclude by discussing implications of our findings for theoretical perspectives and future research on the predictors of and psychological characteristics predicted by hope and optimism. Prior claims about what predicts hope have been mostly supported, but with two additional nuances. First, Bernardo’s (2010) external hope locus and Herth’s (2000) affiliative-contextual dimension have been shown to be important distinctions in understanding how predictors of hope states depend on what is hoped for. Further research may unearth additional characteristics of the objects of hope that moderate predictors’ effects.

The second nuance arises from the findings that greater impairment of quality of life and higher stress scores positively predicted hopes for gaining new skills. These suggest two possible mechanisms. First, particular kinds of challenges may generate specific kinds of hopes oriented to coping with the challenges. Taken together with the Bruininks and Malle (2005) and Bury et al. (2016) materials, a Yerkes-Dodson type of curvilinear relationship between severity of challenge and hope seems plausible. Second, these same challenges may open opportunities and time for gaining new skills and increasing adaptability, and the attractiveness of these opportunities may be heightened by the threat. These interpretations are in line with literature suggesting that uncertain threats motivate striving for compensatory realizations of ideals and the goals associated with them (e.g., McGregor et al., 2010).

Theories about what hopes predict may need revision, guided by two findings in our study. First, that higher scores on hope-optimism regarding skills predicted poorer mental health appears to go against Fredrickson’s broaden-and-build theory. The same is true of connectedness hope scores positively predicting subsequent loneliness scores. The broaden-and-build perspective may need to incorporate scope conditions, for instance regarding the consequences of hope when hopes are dashed. Alternatively, the affective component of hope may not always be experienced as a positive emotion. A Rohingya refugee, interviewed by a journalist about his survival and prospects, remarked that “Hope is torture. It has tortured me” (Huynh, 2020).

The second key finding is prior mental health states moderating the effect of hope state on future mental health states. Our findings suggest that hope has greatest impact on an aspect of mental health (e.g., depression or anxiety) when that aspect is not being experienced at intensive levels. The Arnau et al. (2007) longitudinal study included autoregressive paths taking prior anxiety and depression into account, but they did not test for moderator effects as we have. Our findings may provide a clue to why the evidence thus far regarding whether raising hope has positive effects on mental health is inconclusive [e.g., the review by Schrank et al. (2008)].

Our final recommendation is for more longitudinal studies of state hope and optimism, with more than two measurement occasions for hope and/or optimism indicators. The interplay between hope and other psychological covariates demonstrated in our study begs for investigation and elaboration. Cross-sectional studies cannot deliver this. Hope and optimism often are temporary and even fleeting. They are inherently embedded in psychosocial processes and need to be studied as such.

## Data Availability Statement

The raw data supporting the conclusions of this article will be made available by the authors, without undue reservation.

## Ethics Statement

The studies involving human participants were reviewed and approved by The Australian National University Human Research Ethics Committee. Written informed consent for participation was not required for this study in accordance with the national legislation and the institutional requirements.

## Author Contributions

MS, YS, AD, AC, and NC: methodology, investigation, data curation, and formal analysis. AD and NC: survey administration and supervision. MS: software. MS, AD, and LF: original draft. All authors: conceptualization and reviewing and editing.

## Conflict of Interest

The authors declare that the research was conducted in the absence of any commercial or financial relationships that could be construed as a potential conflict of interest.

## Publisher’s Note

All claims expressed in this article are solely those of the authors and do not necessarily represent those of their affiliated organizations, or those of the publisher, the editors and the reviewers. Any product that may be evaluated in this article, or claim that may be made by its manufacturer, is not guaranteed or endorsed by the publisher.
