# Supplementary material for: The Psychological Benefits of an Uncertain World: Hope and Optimism in the Face of Existential Threat
Source: Front Psychol. 2022 Mar 23;13:749093. doi: 10.3389/fpsyg.2022.749093 (PMC8983926; doi:10.3389/fpsyg.2022.749093)
Supplement: Supplementary file 1 [file Data_Sheet_1.PDF]

The psychological benefits of an uncertain world: Hope and optimism in the face of existential threat: Supplementary material

Michael Smithson, Yiyun Shou, Amy Dawel, Alison Calear, Louise Farrer, Nicolas Cherbuin

All analyses were conducted in R 3.6.3 (R Core Team, 2020), using the cplm (Zhang, 2013), lme4 (Bates, et al., 2015), and ordinal (Christensen, 2019) packages, and Stata 15 (StataCorp, 2017). Table S1 presents summaries of the demographic characteristics of the sample across the six survey waves.

Table S1. Demographic characteristics across survey waves

Wave (date)	1 (28 Mar)	2 (11 Apr)	3 (25 Apr)	4 (09 May)	5 (23 May)	6 (06 June)
Covid cases	3,640	6,292	6,695	6,929	7,106	7,255
N	1296	969	952	910	874	820
Age	46.04 (17.26)	48.56 (16.67)	49.28 (16.32)	49.59 (16.36)	49.84 (16.26)	50.03 (16.09)
Gender						
Male	645 (49.8%)	489 (50.5%)	479 (50.3%)	456 (50.1%)	445 (50.9%)	428 (52.2%)
Female	649 (50.1%)	478 (49.3%)	472 (49.6%)	454 (49.9%)	427 (48.9%)	392 (47.8%)
Other	2 (0.2%)	2 (0.2%)	1 (0.1%)	0 (0%)	2 (0.2%)	0 (0%)
Tertiary ed.	483 (37.6%)	361 (37.5%)	350 (37%)	316 (34.9%)	309 (35.6%)	288 (35.3%)

Table S2 displays the internal consistencies of the scales used in this paper, for each of the relevant survey waves. All of these indicate adequate levels of internal consistency, and these levels are maintained across multiple survey waves.

Table S2. Scale Cronbach's alphas

wave	anxiety	INQ	depression	distress	imp.q.life	loneliness	wellbeing
1	0.941	0.889	0.917	0.922	0.731	0.718	0.914
2	0.945		0.926	0.928	0.767	0.734	
3	0.946		0.925	0.934	0.766	0.756	agency
4	0.944		0.925	0.929	0.794	0.757	0.866
5	0.943		0.923	0.934	0.798	0.777	
6	0.944		0.927	0.934	0.868	0.793	

Figure S1 displays histograms of these scales (they are the means of their respective items with the lowest possible score subtracted from it to produce lower bounds of 0). It is clear that all of them except for loneliness are lower-boundary-inflated. The boundary-inflation is effectively modelled by compound Poisson-gamma (Tweedie) distributions.

Anxiety:

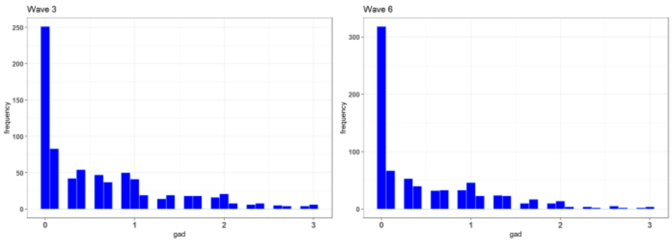

Depression:

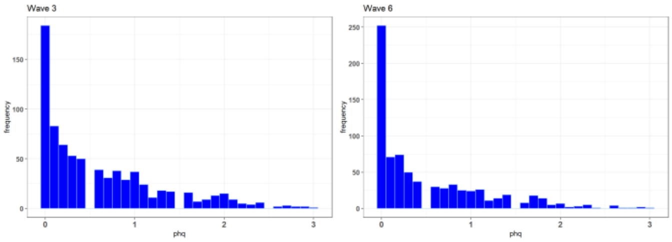

Distress:

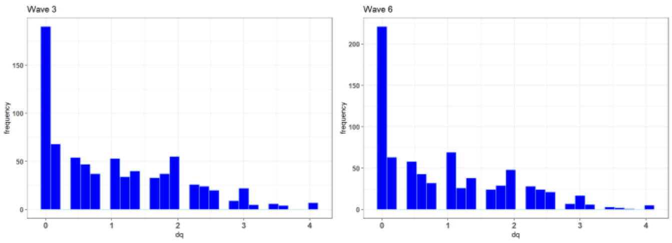

Loneliness:

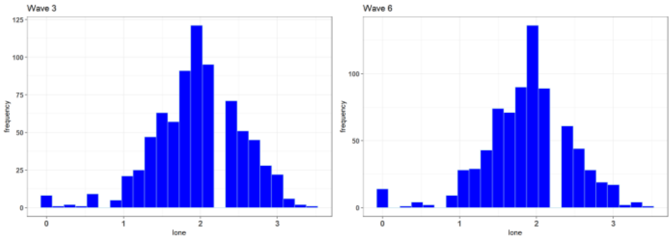

Impaired quality of life:

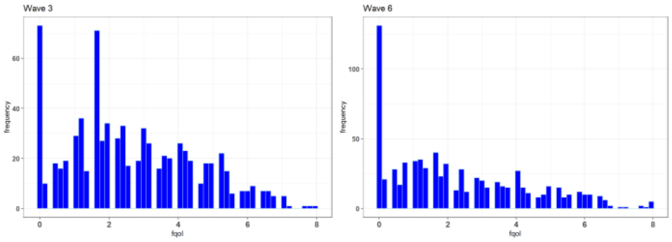

Figure S1. Distributions of mental health covariates, waves 3 and 6

Predictors of Hope-Optimism Items

The models in this section are ordinal logistic regressions with a random intercept, with wave 1 covariates predicting HO items at wave 2 and wave 4 covariates predicting HO items at wave 5. A main goal here is finding out whether covariates at waves 1 and 4 predict HO items similarly at waves 2 and 5, i.e., testing stationarity. If their effects in both pairs of waves are similar, then a model with wave-by-HO items should not significantly improve fit over a model with moderator effects for HO item. Throughout this supplementary document, we will refer to the HO items with the names listed below (the attitude statements are listed after them):

- Skills: You will have new abilities or skills
- Mental Health: Your mental health will have improved
- Connectedness: You will be more connected with people you care about
- Societal Improvement: Our society will have improved in one or more ways

The rating scales had four categories: 1=Not at all ; 2=Slightly hopeful ; 3=Moderately hopeful ; 4=Very hopeful.

Demographics Effects

In our models, education was recoded to years of education. Prior medical and psychological conditions were counts of self-reported conditions selected from a list of them at wave 1. The best model for the demographics variables has the effects of gender, age, and education moderated by HO item, and prior psychological and medical trauma effects moderated by wave-by-HO item. A model with all of the demographic variables' effects moderated by wave-by-HO item does not significantly improve fit (χ_{20}^2 = 14.70, p = .793), whereas a model that removes the wave moderator from the prior psychological and medical trauma effects significantly worsens model fit (χ_{11}^2 = 24.27, p = ..012).

Table S3. Simple-effects for age and education by hope-optimism item

age:

HO item	Coef.	Std. Err.	*z*	*p*	odds-ratio	95%	CI
Skills	-0.055	0.007	-8.23	0.000	0.946	0.934	0.959
Mental Health	-0.024	0.005	-4.48	0.000	0.976	0.966	0.986
Connectedness	0.005	0.006	0.88	0.379	1.005	0.994	1.016
Soc. Improve.	0.012	0.006	2.17	0.030	1.012	1.001	1.024

education:

HO item	Coef.	Std. Err.	*z*	*p*	odds-ratio	95%	CI
Skills	0.276	0.101	2.74	0.006	1.317	1.082	1.604
Mental Health	0.054	0.083	0.65	0.514	1.056	0.897	1.242
Connectedness	-0.060	0.085	-0.71	0.479	0.941	0.797	1.112
Soc. Improve.	0.015	0.087	0.17	0.863	1.015	0.856	1.204

Gender has no significant effects in any of the HO items ($|z| < 1.63$, $p > .103$). Table S3 displays the effects for age and education, showing that there are opposite-signed significant effects for age and just one significant effect for education. The odds-ratios in Table S3 and

subsequent tables reporting ordinal logistic regression results are the coefficients exponentiated. For instance, the Skills coefficient from age is -0.055, and exp(-0.055) = 0.946, so for every additional year of age the odds of hoping for acquiring new skills decreases by a factor of 0.946. The 95% CIs are confidence intervals for the odds-ratios.

Wellbeing and Belonging

The effects of wellbeing and belonging (both at wave 1) were moderated only by HO item. A model that included a wave effect for these two covariates did not significantly improve fit over a model including only moderation by HO item ($\chi_8^2 = 4.87$, $p = .771$).

Table S4. Simple-effects for age and education by HO item

wellbeing:

HO item	Coef.	Std. Err.	*z*	*p*	odds-ratio	95% CI	
Skills	0.092	0.019	4.77	0.000	1.096	1.056	1.139
Mental Health	0.043	0.016	2.72	0.007	1.044	1.012	1.077
Connectedness	-0.005	0.016	-0.32	0.748	0.995	0.964	1.027
Soc. Improve.	0.026	0.017	1.59	0.111	1.027	0.994	1.060

belonging:

HO item	Coef.	Std. Err.	*z*	*p*	odds-ratio	95% CI	
Skills	0.028	0.010	2.92	0.004	1.029	1.009	1.048
Mental Health	0.039	0.008	4.82	0.000	1.040	1.024	1.057
Connectedness	0.096	0.009	10.76	0.000	1.101	1.082	1.120
Soc. Improve.	0.086	0.009	9.62	0.000	1.089	1.070	1.108

Table S4 displays simple-effects results for both covariates. All of the significant effects are positive. Wellbeing has significant effects for Skills and Mental Health, whereas belonging significantly positively predicts all four HO items (most strongly for Connectedness and Societal Improvement).

Mental Health Covariates

Coping had a significant positive effect (z = 4.12, p < .0005; odds-ratio = 1.119, 95% CI = [1.061, 1.180]). A model including moderator effects from HO item and wave did not significantly improve fit (χ_4^2 = 4.65, p = .325).

Anxiety had a significant negative main effect (z = -2.66, p = .008; odds-ratio = 0.972, 95% CI = [0.953, 0.993]). A model with HO item as moderator of the anxiety effect did not significantly improve fit over the main-effect model (χ_3^2 = 6.23, p = .101), and a model with wave as a moderator did not significantly improve fit (χ_1^2 = 1.77, p = .183).

Depression effects were moderated by HO item; this model significantly improved fit over the main-effect model (χ_3^2 = 9.57, p = .023). However, a model with wave as a moderator did not significantly improve fit (χ_1^2 = 0.58, p = .447). Table S5 displays the effects on each HO item.

Distress had significant main and HO-item moderator effects (χ_4^2 = 23.22, p < .0005). A wave-by-item model did not significantly improve fit (χ_3^2 = 1.51, p = 679). Table S5 displays the effects on each HO item.

Impaired quality of life effects with HO item as moderator significantly improved fit over a main-effect model (χ_3^2 = 32.60, p < .0005), but a model with wave as a moderator did not significantly improve fit (χ_1^2 = 1.39, p = .239). Table S5 displays the effects on each HO item.

Loneliness: effects were moderated by HO item; this model significantly improved fit over the main-effect model (χ_3^2 = 29.00, p < .0005), but a model with wave as a moderator did not significantly improve fit (χ_1^2 = 0.12, p = .724). Table S5 displays the effects on each HO item.

Table S5. Simple-effects for depression, distress, loneliness, and impaired quality of life by HO item

depression:

HO item	Coef.	Std. Err.	*z*	*p*	odds-ratio	95% CI	
Skills	-0.021	0.016	-1.27	0.205	0.979	0.948	1.011
Mental Health	-0.032	0.014	-2.25	0.025	0.968	0.942	0.996
Connectedness	-0.055	0.015	-3.64	0.000	0.947	0.919	0.975
Soc. Improve.	-0.056	0.015	-3.67	0.000	0.946	0.918	0.974

distress:

HO item	Coef.	Std. Err.	*z*	*p*	odds-ratio	95% CI	
Skills	-0.012	0.020	-0.6	0.550	0.988	0.950	1.028
Mental Health	-0.005	0.017	-0.28	0.781	0.995	0.962	1.030
Connectedness	-0.046	0.019	-2.51	0.012	0.955	0.921	0.990
Soc. Improve.	-0.038	0.019	-2.05	0.041	0.962	0.928	0.998

impaired quality of life:

HO item	Coef.	Std. Err.	*z*	*p*	odds-ratio	95% CI	
Skills	0.043	0.010	4.51	0.000	1.044	1.025	1.063
Mental Health	0.010	0.008	1.21	0.228	1.010	0.994	1.026
Connectedness	0.012	0.009	1.45	0.148	1.012	0.996	1.030
Soc. Improve.	0.002	0.009	0.21	0.836	1.002	0.985	1.019

loneliness:

HO item	Coef.	Std. Err.	*z*	*p*	odds-ratio	95% CI	
Skills	-0.089	0.021	-4.23	0.000	0.915	0.878	0.953
Mental Health	-0.067	0.018	-3.84	0.000	0.935	0.904	0.968
Connectedness	-0.123	0.018	-6.66	0.000	0.884	0.853	0.917
Soc. Improve.	-0.121	0.018	-6.58	0.000	0.886	0.855	0.919

Lack of agency scores negatively predicted all HO items in wave 5, as Table S6 shows. The strongest effects were for the Connectedness and Societal Improvement items.

Table S6. Simple-effects for mastery by HO item

HO item	Coef.	Std. Err.	*z*	*p*	odds-ratio	95%	CI
Skills	-0.343	0.102	-3.37	0.001	0.710	0.581	0.866
Mental Health	-0.434	0.098	-4.44	0.000	0.648	0.535	0.784
Connectedness	-0.566	0.097	-5.86	0.000	0.568	0.470	0.686
Soc. Improve.	-0.600	0.096	-6.26	0.000	0.549	0.455	0.662

Stress: Stress effects exhibited significant moderation by HO item ($\chi_4^2 = 12.56$, $p = .014$) and by wave ($\chi_1^2 = 4.79$, $p = .028$), but with no significant wave-by-item effect ($\chi_3^2 = 3.26$, $p = .354$). Table S7 displays the relevant parts of the output from the random-intercepts ordinal logistic regression model.

Table S7. Relevant output for the stress model

wave	HO item	Coef.	Std. Err.	*z*	*p*	odds-ratio	95%	CI
2	Skills	0.014	0.055	0.25	0.804	1.014	0.910	1.129
2	Mental Health	-0.019	0.053	-0.36	0.716	0.981	0.884	1.088
2	Connectedness	-0.032	0.053	-0.61	0.540	0.968	0.874	1.073
2	Soc. Improve.	-0.128	0.052	-2.45	0.014	0.880	0.795	0.975
5	Skills	0.108	0.052	2.07	0.038	1.114	1.006	1.234
5	Mental Health	0.075	0.050	1.50	0.135	1.078	0.977	1.189
5	Connectedness	0.062	0.055	1.26	0.209	1.064	0.955	1.185
5	Soc. Improve.	-0.033	0.049	-0.68	0.497	0.967	0.878	1.065

Covariates predicted by hope-optimism items

This section provides details about the models assessing whether the hope-optimism items predict mental health and consequences variables.

Mental Health Covariates

Coping was positively predicted by the Skills (z = 2.512, p = .012) and Societal Improvement item (z = 2.691, p = .007), but not significantly by the other HO items ($z \leq$ 0.966, p $\geq$ .334). Moderator effects from prior coping were significantly negative for both Skills (z = -2.124, p = .034) and Societal Improvement item (z = -2.073, p = .038). Table S8 displays the relevant parts of the output from the random-intercepts linear regression model.

Table S8. Relevant output for the coping model

Covariate	Coef.	Std. Err	*z*	*p*	95% CI	
wave	0.135	0.093	1.451	0.147	-0.047	0.317
lag	1.139	0.109	10.472	< .0005	0.925	1.353
Skills	0.470	0.187	2.513	0.012	0.103	0.837
Mental Health	-0.022	0.193	-0.112	0.911	-0.400	0.356
Connectedness	0.190	0.196	0.966	0.334	-0.194	0.574
Societal Improvement	0.512	0.190	2.691	0.007	0.140	0.884
lag*Skills	-0.096	0.045	-2.124	0.034	-0.184	-0.008
lag*Mental Health	0.026	0.048	0.543	0.587	-0.068	0.120
lag*Connectedness	-0.050	0.049	-1.026	0.305	-0.146	0.046
lag*Soc. Improvement	-0.097	0.047	-2.073	0.038	-0.189	-0.005

Note: lag = prior coping

Anxiety required an anxiety-by-wave model but showed stationarity because a model including wave-by-HO-item terms did not significantly improve model fit over a model without this term (χ_4^2 = 3.462, p = .484). Table S9 displays the relevant parts of the output

from the random-intercepts compound Poisson-gamma (Tweedie) GLM. The coefficients for this type of GLM may be interpreted in the same way as unstandardized linear regression coefficients.

Table S9. Relevant output for the anxiety model

Covariate	Coef.	Std. Err	*z*	*p*	95%	CI
lag	0.893	0.08	11.175	< .0005	0.736	1.050
wave	-0.283	0.058	-4.903	< .0005	-0.397	-0.169
Skills	0.231	0.084	2.737	0.006	0.066	0.396
Mental Health	0.143	0.087	1.646	0.100	-0.028	0.314
Connectedness	-0.038	0.091	-0.417	0.677	-0.216	0.140
Societal Improvement	-0.312	0.085	-3.658	< .0005	-0.479	-0.145
lag*wave	0.108	0.025	4.279	< .0005	0.059	0.157
lag*Skills	-0.131	0.04	-3.302	0.001	-0.209	-0.053
lag*Mental Health	-0.043	0.04	-1.089	0.276	-0.121	0.035
lag*Connectedness	0.025	0.042	0.594	0.553	-0.057	0.107
lag*Soc. Improvement	0.145	0.038	3.776	< .0005	0.071	0.219

Note: lag = prior anxiety

In this paper we claim that the Skills HO item more weakly positively predicted anxiety as prior anxiety increased, whereas the Societal Improvement item more weakly negatively predicted anxiety as prior anxiety increased. Similar moderator effects are reported for other mental health variables in this subsection, so here we provide an explanation of this type of effect, using the Table S7 results as an example. Starting with the Skills item, its coefficient is positive (0.231) and the moderator effect coefficient is negative (-0.131). Therefore, as prior anxiety (lag) increases the net effect of Skills on subsequent anxiety decreases. Turning to the Societal Improvement item, its coefficient is negative (-0.312) but its moderator effect

is positive (0.145). Thus, as prior anxiety (lag) increases the net effect of Skills on subsequent anxiety increases.

Depression required a wave-by-depression model, but showed stationarity in the HO-item effects because a model including wave-by-HO-item terms did not significantly improve model fit over a model without this term (χ_4^2 = 1.779, *p* = .776). All of the depression-by-HO-item terms are significant but do not have the same signs. The Skills and Mental Health HO items' coefficients for depression decreased as prior depression increased, whereas the Connectedness and Societal Improvement items' coefficients for depression increased as prior depression increased. Table S10 displays the relevant parts of the output from the random-intercepts compound Poisson-gamma (Tweedie) GLM.

Table S10. Relevant output for the depression model

Covariate	Coef.	Std. Err	*z*	*p*	95%	CI
lag	0.825	0.078	10.613	< .0005	0.672	0.978
wave	-0.312	0.055	-5.660	< .0005	-0.420	-0.204
Skills	0.123	0.080	1.538	0.124	-0.034	0.280
Mental Health	0.193	0.085	2.288	0.022	0.026	0.360
Connectedness	-0.254	0.090	-2.814	0.005	-0.430	-0.078
Societal Improvement	-0.162	0.084	-1.921	0.055	-0.327	0.003
lag*wave	0.125	0.025	4.923	< .0005	0.076	0.174
lag*Skills	-0.103	0.039	-2.609	0.009	-0.179	-0.027
lag*Mental Health	-0.084	0.042	-2.027	0.043	-0.166	-0.002
lag*Connectedness	0.140	0.045	3.129	0.002	0.052	0.228
lag*Soc. Improvement	0.086	0.041	2.094	0.037	0.006	0.166

Note: lag = prior depression

Distress did not require a distress-by-wave model (χ_1^2 = 2.173, *p* = .141), and showed stationarity because a model including wave-by-HO-item terms did not significantly improve model fit over a model without this term (χ_4^2 = 3.556, *p* = .469). The Skills HO item more

weakly positively predicted distress as prior distress increased, whereas the Societal Improvement item more generally negatively predicted distress. The Mental Health and Connectedness items do not have significant effects. Table S11 displays the relevant parts of the output from the random-intercepts compound Poisson-gamma (Tweedie) GLM.

Table S11. Relevant output for the distress model

Covariate	Coef.	Std. Err.	*z*	*p*	95%	CI
lag	0.556	0.046	12.001	< .0005	0.466	0.646
wave	-0.011	0.018	-0.583	0.560	-0.046	0.024
Skills	0.093	0.066	1.421	0.156	-0.036	0.222
Mental Health	0.102	0.068	1.507	0.132	-0.031	0.235
Connectedness	-0.085	0.069	-1.233	0.218	-0.220	0.050
Societal Improvement	-0.266	0.066	-4.039	< .0005	-0.395	-0.137
lag*Skills	-0.055	0.023	-2.329	0.020	-0.100	-0.010
lag*Mental Health	-0.025	0.024	-1.041	0.298	-0.072	0.022
lag*Connectedness	0.030	0.025	1.193	0.233	-0.019	0.079
lag*Soc. Improvement	0.086	0.023	3.709	< .0005	0.041	0.131

Note: lag = prior distress

Loneliness did not require a loneliness-by-wave model (χ_1^2 = 1.368, p = .242), and showed stationarity because a model including wave-by-HO-item terms did not significantly improve model fit over a model without this term (χ_4^2 = 8.410, p = .078). The Skills and Connectedness items more weakly positively predicted loneliness as prior loneliness increased. The Mental Health and Societal Improvement items do not have significant effects. Table S12 displays the relevant parts of the output from the random-intercepts linear regression.

Table S12. Relevant output for the loneliness model

Covariate	Coef.	Std. Err.	*z*	*p*	95%	CI
lag	0.900	0.047	19.190	< .0005	0.808	0.992
wave	-0.021	0.012	-1.785	0.075	-0.045	0.003
Skills	0.158	0.053	3.002	0.003	0.054	0.262
Mental Health	-0.066	0.054	-1.212	0.226	-0.172	0.040
Connectedness	0.135	0.055	2.468	0.014	0.027	0.243
Societal Improvement	-0.065	0.056	-1.161	0.246	-0.175	0.045
lag*Skills	-0.070	0.021	-3.404	0.001	-0.111	-0.029
lag*Mental Health	0.035	0.021	1.657	0.098	-0.006	0.076
lag*Connectedness	-0.052	0.021	-2.489	0.013	-0.093	-0.011
lag*Soc. Improvement	0.002	0.021	0.078	0.938	-0.039	0.043

Note: lag = prior loneliness

Impaired quality of life required a impaired quality of life-by-wave model, but showed stationarity for the HO-item effects because a model including wave-by-HO-item terms did not significantly improve model fit over a model without this term (χ_4^2 = 3.175, p = .529). The Mental Health HO item negatively predicted impaired quality of life. The other three HO items have significant impaired quality of life-by-HO-item terms. The Skills and Connectedness items positively predicted impaired quality of life, but more weakly as prior impaired quality of life increased, whereas the Societal Improvement item negatively predicted impaired quality of life, but more weakly as prior impaired quality of life increased. Table S13 displays the relevant parts of the output from the random-intercepts compound Poisson-gamma (Tweedie) GLM.

Table S13. Relevant output for the impaired quality of life model

Covariate	Coef.	Std. Err.	*z*	*p*	95%	CI
lag	0.266	0.023	11.463	< .0005	0.221	0.311
wave	-0.117	0.040	-2.923	0.004	-0.195	-0.039
Skills	0.140	0.052	2.665	0.008	0.038	0.242
Mental Health	-0.118	0.055	-2.138	0.033	-0.226	-0.010
Connectedness	0.147	0.058	2.556	0.011	0.033	0.261
Societal Improvement	-0.120	0.056	-2.145	0.032	-0.230	-0.010
lag*wave	0.024	0.008	2.932	0.003	0.008	0.040
lag*Skills	-0.024	0.011	-2.146	0.032	-0.046	-0.002
lag*Mental Health	0.021	0.012	1.772	0.077	-0.003	0.045
lag*Connectedness	-0.032	0.012	-2.613	0.009	-0.056	-0.008
lag*Soc. Improvement	0.029	0.012	2.423	0.016	0.005	0.053

Note: lag = prior impaired quality of life

Consequences

Random-intercepts ordinal logistic (cumulative logit) models were used for testing whether hope items in wave 2 predict the consequence item ratings in wave 5. A wave-by-HO-item terms significantly improved model fit over a model without this term (χ_4^2 = 85.891, $p < .0005$). The Skills HO item positively predicted all of the consequence item ratings ($z \geq 3.15$, $p < .002$). The Mental Health item positively predicted the recreation consequence item ($z = 3.13$, $p = .002$); the Connectedness item positively predicted the adaptation ($z = 2.09$, $p = .037$), skills ($z = 2.12$, $p = .034$), and relationships ($z = 5.09$, $p < .0005$) consequence items; and the Societal Improvement item positively predicted the coping consequence item ($z = 2.27$, $p = .023$). Table S14 displays the relevant parts of the

output from the final model. The coefficients are those obtained by using each consequence item as the base group in the model.

Table S14. Relevant output for the consequences model

HO item	Coef.	Std. Err.	*z*	*p*	odds-ratio	95%	CI
Privacy							
Skills	0.421	0.128	3.28	0.001	1.523	1.185	1.958
Mental Health	0.111	0.129	0.86	0.388	1.117	0.868	1.439
Connectedness	0.222	0.130	1.70	0.089	1.249	0.968	1.611
Soc. Improvemt.	-0.053	0.130	-0.41	0.683	0.948	0.735	1.224
Legal							
Skills	0.589	0.129	4.58	< .0005	1.802	1.400	2.321
Mental Health	0.202	0.128	1.58	0.115	1.224	0.952	1.573
Connectedness	0.005	0.130	0.04	0.967	1.005	0.779	1.297
Soc. Improvemt.	-0.140	0.128	-1.09	0.277	0.869	0.676	1.117
Adaptation							
Skills	0.483	0.125	3.86	< .0005	1.621	1.269	2.071
Mental Health	0.095	0.125	0.76	0.447	1.100	0.861	1.405
Connectedness	0.263	0.126	2.09	0.037	1.301	1.016	1.665
Soc. Improvemt.	0.099	0.125	0.79	0.431	1.104	0.864	1.411
Coping							
Skills	0.558	0.126	4.42	< .0005	1.747	1.365	2.237
Mental Health	-0.074	0.126	-0.59	0.557	0.929	0.725	1.189
Connectedness	0.223	0.126	1.77	0.076	1.250	0.976	1.600
Soc. Improvemt.	0.287	0.126	2.27	0.023	1.332	1.041	1.706
Skills							

Skills	0.547	0.126	4.34	< .0005	1.728	1.350	2.212
Mental Health	0.113	0.126	0.89	0.371	1.120	0.875	1.433
Connectedness	0.269	0.127	2.12	0.034	1.309	1.020	1.679
Soc. Improvemt.	0.100	0.126	0.79	0.427	1.105	0.863	1.415
Relations							
Skills	0.410	0.130	3.15	0.002	1.507	1.168	1.944
Mental Health	0.049	0.131	0.38	0.705	1.050	0.812	1.358
Connectedness	0.673	0.132	5.09	< .0005	1.960	1.513	2.539
Soc. Improvemt.	-0.040	0.129	-0.31	0.755	0.961	0.746	1.237
Recreation							
Skills	0.747	0.130	5.73	< .0005	2.111	1.636	2.723
Mental Health	0.404	0.129	3.13	0.002	1.498	1.163	1.929
Connectedness	0.056	0.130	0.43	0.668	1.058	0.820	1.365
Soc. Improvemt.	-0.125	0.130	-0.96	0.336	0.882	0.684	1.139

References

Bates, D., Maechler, M., Bolker, B, & Walker, S. (2015). Fitting Linear Mixed-Effects Models Using lme4. *Journal of Statistical Software*, 67(1), 1-48.

Christensen, R. H. B. (2019). *ordinal - Regression Models for Ordinal Data*. R package version 2019.12-10. https://CRAN.R-project.org/package=ordinal.

R Core Team (2020). *R: A language and environment for statistical computing*. R Foundation for Statistical Computing, Vienna, Austria. URL https://www.R-project.org/.

StataCorp. (2017). *Stata Statistical Software: Release 15*. College Station, TX: StataCorp LLC.

Zhang, Y. (2013). Likelihood-based and Bayesian Methods for Tweedie Compound Poisson Linear Mixed Models. *Statistics and Computing*, 23, 743-757.
